# Pelvic Floor Muscle Training Following Surgery for Pelvic Organ Prolapse: Recommendation from Scientific Literature

**DOI:** 10.3390/jcm15031116

**Published:** 2026-01-30

**Authors:** Gianfranco Lamberti, Donatella Giraudo, Gianluca Ciardi, John Kenneth Levis

**Affiliations:** 1 Department of Medicine and Surgery, University of Parma, 43121 Parma, Italy; 2 Department of Physical Medicine and Rehabilitation, AUSL Piacenza, 29121 Piacenza, Italy; gianluca.ciardi@unipr.it; 3 Department of Urology, San Raffaele Turro Hospital, 20132 Milano, Italy; giraudo.donatella@hsr.it; 4 University of Parma—Department of Medicine and Surgery-Degree Course in Physiotherapy—Piacenza Training Centre, 29017 Fiorenzuola d’Arda, Italy; 5 Postgraduate School of Physical and Rehabilitation Medicine, University of Parma, 43121 Parma, Italy; johnkennethbrian.levis@unipr.it

**Keywords:** pelvic organ prolapse, POP, pelvic floor muscle training, PFMT

## Abstract

**Background:** POP surgery improves anatomical support and quality of life, but urinary, bowel, sexual, and pain issues are common after surgery. The role of rehabilitation in addressing these problems is recognized, though not yet clearly defined. **Objective:** This scoping review aims to map the clinical evidence on conservative rehabilitation interventions for urinary, bowel, and sexual dysfunction, and pelvic pain after POP surgery. **Methods:** In accordance with PRISMA-ScR guidelines, we included randomized controlled trials, cohort studies, observational studies, and systematic reviews relevant to post-surgical rehabilitation options frequently encountered in clinical settings, including pelvic floor muscle training (PFMT), physiotherapy, and multimodal programs. Meta-analysis was not conducted due to clinical and methodological heterogeneity across the studies. **Results:** PFMT demonstrates beneficial effects on symptom severity and pelvic muscle function in women with POP. Postoperative rehabilitation may improve urinary continence, sexual function, and pelvic pain, although the strength of current evidence remains limited. Many studies prioritize surgical revision over conservative management, and the effectiveness of rehabilitation for persistent or de novo symptoms is not well established. **Conclusions:** Conservative rehabilitation, especially PFMT, may aid recovery and improve function after POP surgery. More research is needed to define the optimal protocols and to determine how to incorporate them into post-surgical care.

## 1. Introduction

Pelvic organ prolapse (POP) refers to the descent of one or more pelvic organs—uterus, vagina, bladder, or bowel—through the vaginal canal. Prolapse is categorized as apical (uterine or post-hysterectomy vault), anterior (cystocele, urethrocele, or paravaginal defect), or posterior (enterocele, rectocele, or perineal defect). POP is prevalent in postmenopausal women and frequently involves multiple compartments simultaneously.

In the early stages, many women have no symptoms. As prolapse worsens, pelvic pressure, discomfort, and organ-specific issues affect daily activities and quality of life.

From a pathophysiological standpoint, pelvic organ prolapse is the ultimate manifestation of a progressive failure in the pelvic support structures, involving both the pelvic floor muscles and connective tissue. The International Continence Society (ICS) recommends that evaluating pelvic floor health should include standardized tests of muscle strength, endurance, coordination, and relaxation, along with a systematic examination of pelvic organ support using the POP-Q system [1]. This thorough approach helps clinicians link structural issues with functional impairments and symptoms.

Apart from digital exams, pelvic floor ultrasound—especially transperineal and endovaginal scans—has become an important method for assessing levator ani strength, hiatal size, organ mobility, and connective tissue defects. Ultrasound allows objective visualization of muscle injury, avulsions, and support problems that are closely linked to prolapse development, recurrence, and postoperative problems [2,3].

The biomechanical model called Petros’ Integral Theory explains how tissue damage relates to clinical symptoms. It suggests that prolapse and related urinary, bowel, sexual, and pain issues stem from weakened ligament support and altered neuromuscular control of the pelvic floor [4]. Surgery can fix anatomy but does not automatically restore nerve and muscle function. Hence, postoperative rehabilitation, especially pelvic floor muscle training, is crucial for re-establishing proper muscle coordination, load sharing, and continence within the support system.

This comprehensive understanding of anatomy, neuromuscular control, and tissue mechanics underpins why rehabilitation is an essential part of managing women undergoing pelvic organ prolapse surgery.

Pelvic floor muscle training (PFMT) is the primary conservative management option for women with symptomatic POP. PFMT aims to strengthen, coordinate, and enhance the endurance of pelvic floor muscles, which can improve the support of pelvic organs and alleviate symptoms such as incontinence and pelvic discomfort. Success depends on accurate assessment, supervised exercise instruction, and patient adherence, with studies showing symptom improvement and enhanced functional capacity [5,6].

Treatment depends on prolapse stage, symptoms, and patient preference, and includes conservative approaches—like observation, pessaries, and PFMT—or surgery. Surgery can be vaginal, laparoscopic, robotic, or open, with or without mesh. Choice depends on patient expectations, reproductive plans, and goals. Both methods aim to restore support and reduce symptoms [7].

About 11% of women will have POP surgery by age 79 [8]. This risk may double in the next 20 years due to aging.

Despite the anatomical success of prolapse repair, many women experience recurrence or new-onset pelvic floor dysfunctions after surgery. Rates of anatomical recurrence range from 19% to 45%. Reoperation rates reach 3–20%, depending on the surgical technique and length of follow-up [9,10,11]. Stress urinary incontinence may persist or newly appear in 4–50% of patients [12,13,14]. De novo overactive bladder symptoms occur in about 5–30% [13,14,15,16]. Dyspareunia and sexual dysfunction are reported in 5–17% of women [12]. New-onset dyspareunia affects about 7% two years after vaginal repair [13,14,16,17]. Bowel dysfunctions such as obstructed defecation persist in up to 37% of cases. Fecal incontinence occurs in up to 5% [16,18,19]. Chronic or new pelvic pain develops in 2.5–17% of cases, especially after mesh-based repairs or sacrospinous fixation [13,16,20,21].

These dysfunctions—whether persistent or new—impact the urinary, bowel, sexual, and pain domains and worsen quality of life. Table 1 presents the main outcomes and rates, highlighting the need for targeted rehabilitation.

Emerging evidence suggests that women with stronger pelvic floor muscle function before surgery may be less likely to have recurrence or require reoperation. This supports PFMT in the perioperative phase, as it has demonstrated benefits such as increased muscle strength and coordination, reduced risk of recurrent urinary, bowel, or sexual dysfunction, and improved quality of life after surgery.

The multidisciplinary management of complications after pelvic reconstructive surgery—especially mesh complications—has been emphasized in the Joint Position Statement from AUGS, SUFU, SGS, and IUGA (2020/2021) [25]. This highlights the value of conservative approaches, such as pelvic floor physical therapy, pain management, and psychological support. It aligns with evidence supporting physiatrically guided rehabilitation as part of conservative management for postoperative pelvic floor dysfunction.

Pelvic surgery may affect the function of pelvic floor muscles, and the connective tissue supporting pelvic organs, leading to dysfunction. PFMT specifically targets strength, endurance, and coordination, which can help reduce symptoms of prolapse, urinary and fecal incontinence, and sexual dysfunction, therefore representing a practical and low-risk support intervention.

Adding PFMT to perioperative care aligns with the Enhanced Recovery After Surgery (ERAS) model, which encourages early mobilization, multimodal management, and faster recovery [26]. These methods aim to improve healing and quality of life beyond anatomy alone.

Despite increasing attention, available literature remains fragmented and lacks a comprehensive synthesis of postoperative rehabilitation strategies following POP surgery.

This review focuses on dysfunctions likely to respond to PFMT—such as incontinence, defecatory disorders, sexual dysfunction, and pelvic pain—not on mechanical issues like retention, mesh exposure, or structural failure, which are outside rehabilitation. The aim is to identify and summarize evidence for PFMT in postoperative rehab for POP in women, examining effects on recovery, quality of life, and recurrence, while highlighting current knowledge gaps and directions for future research and practice. Although the primary focus of this review is postoperative rehabilitation, selected preoperative studies were included to provide a necessary interpretative framework for understanding the timing and potential mechanisms of pelvic floor muscle training. Specifically, preoperative trials allow comparison of neuromuscular training delivered in the presence of distorted pelvic anatomy with training applied after surgical restoration of support. The consistent lack of postoperative benefit following preoperative PFMT observed in high-quality trials suggests that anatomical correction may be a prerequisite for effective neuromuscular re-education. In this way, preoperative evidence directly informs the postoperative context by clarifying the physiological conditions under which rehabilitation is more likely to be effective.

## 2. Materials and Methods

### 2.1. Rationale for Choosing a Scoping Review Design

A scoping review was chosen because of the varied study designs, interventions, and outcomes in the POP rehab literature. Existing research is fragmented and variable, especially regarding bowel function, pain, and sexual health. Included studies range from RCTs to systematic reviews, but outcomes are inconsistently reported. A scoping review helps map the evidence and gaps, and clarifies the range of rehab strategies for POP-related dysfunction. This follows guidance from the Joanna Briggs Institute (JBI) [27] and the PRISMA-ScR checklist [28].

### 2.2. Eligibility Criteria

The eligibility criteria were defined using the Population–Concept–Context (PCC) framework recommended by JBI. The population of interest included women who underwent surgical treatment for pelvic organ prolapse (POP), regardless of surgical approach or stage of prolapse. The concept focused on any form of postoperative rehabilitation intervention, including pelvic floor muscle training (PFMT), biofeedback, electrical stimulation, manual therapy, behavioral education, or multidisciplinary care. The context encompassed all healthcare settings in which postoperative rehabilitation was provided, including inpatient, outpatient, and community-based services, without restriction by geographic location or healthcare system.

Studies were eligible for inclusion if they met the following criteria:

(a) Involved women after POP surgery;
(b) Evaluated at least one form of pelvic floor rehabilitation;
(c) Reported outcomes related to urinary, bowel, sexual function, pain, or recurrence (quality of life outcomes were also considered when available);
(d) Were published in peer-reviewed journals and written in English or French.

Both empirical studies (randomized controlled trials, observational studies, feasibility or pilot studies) and secondary literature (systematic reviews, narrative reviews, expert consensus, clinical reports) were considered eligible for inclusion, reflecting the exploratory aim of this scoping review. Articles without clinical outcome data relevant to the dysfunctions of interest, those reporting exclusively anatomical outcomes, or those lacking methodological transparency were excluded from structured appraisal but could be referenced narratively for context.

### 2.3. Information Sources and Search Strategy

A comprehensive search strategy was developed in consultation with the Joanna Briggs Institute (JBI) methodological guidance for scoping reviews. The following electronic databases were searched: PubMed/MEDLINE, Embase, CINAHL, Scopus, PEDro, and Google Scholar. The PubMed search string combined controlled vocabulary (MeSH terms) and free-text terms related to pelvic organ prolapse, surgery, and rehabilitation. Search strategies were then adapted to the indexing systems of each database (Emtree for Embase, CINAHL Headings for CINAHL, keyword and title/abstract fields for Scopus and PEDro). For Google Scholar, simplified keyword combinations were applied, given the limitations of Boolean operators, and the first 200 results were screened as recommended for scoping reviews. The complete search strategies for all databases are provided in Table S1. No restrictions were applied on language, publication date, or geographic location at the initial search stage.

### 2.4. Screening of Records and Critical Appraisal

All records were manually screened by two independent reviewers, while a third opinion was requested to solve conflicts. After removing duplicates and unreadable records, a first screening for title and abstract was applied. Then, eligible papers were recovered in full text, and by application of inclusion criteria (PCC) relevant articles were selected for the final stage of the review.

Although not even performed for scoping design, we planned a critical appraisal for included papers, in order to inform the interpretation of findings and highlight areas where evidence is more or less robust. Particularly, we used the following:

- Cochrane Risk of Bias 2 (RoB 2) [29] and PEDRO scale tools for RCTs.
- AMSTAR 2 [30] checklist for systematic review.
- SANRA (Scale for the Assessment of Narrative Review Articles) [31] for narrative reviews.
- JBI Checklist for Text and Opinion [32] for expert opinion papers.

## 3. Results

**Figure 1:**
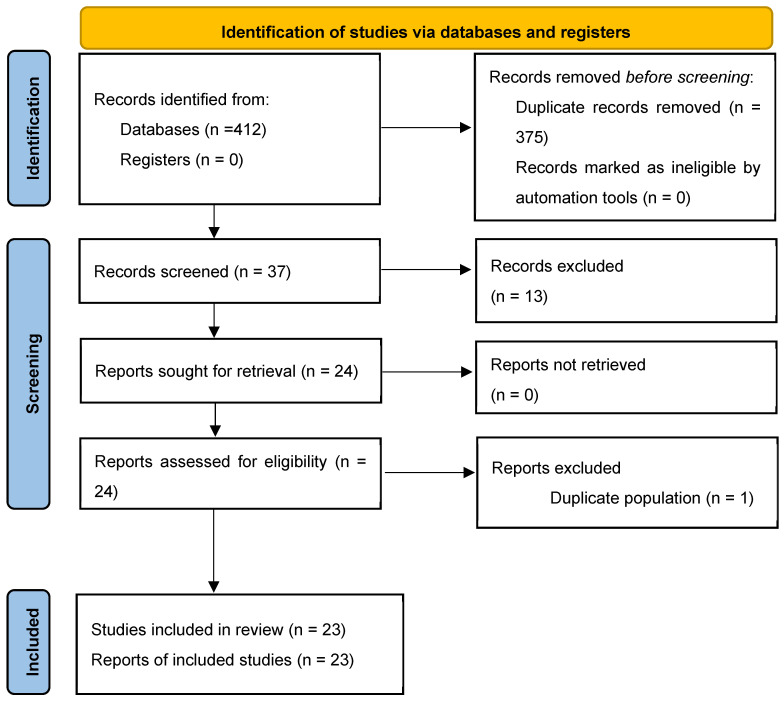
PRISMA-ScR flow diagram of the review.

As shown in Figure 1, the database search yielded 412 records: 65 from PubMed, 98 from Embase, 38 from CINAHL, 11 from PEDro, and 200 from Google Scholar. After removal of duplicates, 37 unique records remained. These were screened by title and abstract, leading to the exclusion of 12 studies that did not meet the eligibility criteria. The full text of the remaining 24 articles was retrieved and assessed for eligibility. Among these, one article was excluded from the quantitative count because it reported on the same study population as another included publication, although both were retained for descriptive purposes as they provided complementary information (e.g., different outcomes or follow-up periods). As a result, 23 studies were included in the final synthesis: 15 randomized controlled trials, 4 systematic reviews, 2 narrative reviews, and 1 expert or opinion paper.

## 4. Appraisal

Sixteen of our included papers [33,34,35,36,37,38,39,40,41,42,43,44,45,46,47] were RCTs, and Rob 2 evaluation was performed. Four systematic reviews [48,49,50,51] were analysed through AMSTAR 2 tool; two narrative reviews [52,53] was examined with SANRA scale, and finally a clinical opinion paper [54] was analysed through JBI text & opinion tool.

Table 2, Table 3, Table 4 and Table 5 summarizes included articles’ main characteristics and appraisal.

### 4.1. Overall Commentary on Randomized Controlled Trials and Reviews Evaluating PFMT After POP Surgery

The randomized and secondary literature on PFMT as an adjunct to surgery for pelvic organ prolapse (POP) provides a broad yet heterogeneous picture. Most studies converge on the feasibility and safety of perioperative rehabilitation but diverge regarding its additive benefit beyond surgical repair.

Dawson et al. (2018) [36] conducted a randomized controlled trial following vaginal reconstructive surgery for POP to evaluate whether an early, structured physiotherapy program—focused on muscle awareness, strength, and coordination—could improve postoperative recovery compared with standard care. Both groups demonstrated significant improvements in pain, urinary symptoms, and quality of life (PFDI-20, PFIQ-7, WHOQOL-BREF) at twelve weeks, with no statistically significant differences between them. Although available only as a conference abstract, this trial supports the feasibility and safety of early postoperative rehabilitation and provides preliminary evidence that structured physiotherapy may facilitate functional recovery.

Barber et al. (2014) [33] examined perioperative behavioral therapy combined with PFMT (BPMT) versus usual care. At two years, BPMT did not significantly improve urinary or prolapse symptom scores (UDI, POPDI) or anatomical outcomes. This high-quality multicenter trial—with masked assessors and robust outcome definitions—offers strong evidence that brief perioperative PFMT confers no measurable benefit when performed alongside surgical correction. Nonetheless, the authors acknowledged that targeted rehabilitation might still be relevant for women with residual or de novo dysfunctions after surgery, encouraging further research in this subgroup.

In a secondary analysis of the same OPTIMAL trial, Borello-France et al. (2023) [34] investigated adherence to BPMT and its association with outcomes at 24 months. Despite standardized therapist training, long-term adherence was poor, and no additional benefit was detected from perioperative PFMT. These findings highlight behavioral and motivational barriers to sustained engagement, suggesting that postoperative rehabilitation requires individualized follow-up, prolonged supervision, and strategies to enhance adherence rather than short perioperative programs.

Brandt and Janse van Vuuren (2022) [35] compared three postoperative strategies in women undergoing reconstructive surgery for stage II–III POP: (1) PFMT alone, (2) PFMT combined with abdominal training using pressure biofeedback and EMG guidance, and (3) standard postoperative counseling. Both PFMT groups, trained twice weekly for six months, achieved superior gains in pelvic muscle strength, endurance, and contraction speed compared with controls. However, quality-of-life (P-QOL) and anatomical (POP-Q) improvements were similar across all arms, indicating that the primary benefit was derived from surgical repair itself. Despite a modest sample and short follow-up, the study confirms that postoperative PFMT is a safe and feasible means of restoring muscular performance, even if its short-term symptomatic advantages remain limited.

Similarly, Duarte et al. (2020) [37] found no added benefit from an intensive PFMT regimen (four preoperative and seven postoperative sessions) over surgery alone. Both groups experienced significant postoperative improvements in prolapse symptoms (PFDI-20), pelvic floor strength, quality of life, and sexual function at 40 and 90 days, with no significant between-group differences.

Frawley et al. (2010) [38] investigated individualized PFMT as an adjunct to prolapse surgery or hysterectomy. Despite 1 preoperative and 7 postoperative sessions over 12 months, no significant between-group differences emerged in symptoms or quality of life. Participants receiving physiotherapy exhibited greater ability to perform correct pelvic floor contractions, but this did not translate into measurable clinical gains. As noted by Bø et al. (2022) [52], the trial provides early evidence of improved muscular control without a clear symptomatic impact during the first postoperative year.

Jarvis et al. (2005) [39] reported more favorable short-term results. In 60 women undergoing corrective surgery for incontinence or POP, perioperative physiotherapy—including PFMT, the “Knack,” and bladder-bowel education—led to greater reductions in urinary symptoms, enhanced quality of life, and stronger pelvic contractions at 12 weeks compared with standard care. However, the trial was small and methodologically limited, and later [50] classified it as a preliminary feasibility study with inconclusive efficacy.

In the long-term follow-up of the OPTIMAL study, Jelovsek et al. (2018) [40] confirmed the lack of any sustained effect of perioperative BPMT. At five years, neither anatomical nor symptomatic outcomes differed significantly between behavioral and standard care groups, effectively ruling out delayed or cumulative benefits of PFMT.

Liang et al. (2019) [41] explored a short-term perioperative PFMT program combining four supervised sessions with daily home exercises. Both groups improved across prolapse-related symptoms, but the PFMT group demonstrated greater reductions in urinary distress (UDI-6) at 42 and 60 days. No differences were noted in POPDI-6 or CRADI-8 scores. These findings suggest that early supervised PFMT may selectively support urinary recovery by reactivating bladder control mechanisms, warranting longer-term studies to confirm its global impact.

McClurg et al. (2014) [43] conducted a feasibility RCT comprising one preoperative and six postoperative sessions over 12 weeks. The PFMT group reported fewer prolapse symptoms and stronger muscle performance at 12 months, confirming safety, acceptability, and potential symptomatic benefit, though the study was not powered to assess efficacy.

Pauls et al. (2014) [45] evaluated a structured postoperative PFMT program (five sessions with EMG feedback). Temporary improvements in EMG activity and muscle control were observed at 12 weeks but were not maintained at 6 months, reinforcing the view that short-term supervised PFMT yields transient neuromuscular gains without long-term anatomical or symptomatic advantages.

Wang et al. (2023) [46] analyzed the interaction between preoperative pelvic pain and postoperative outcomes within the OPTIMAL cohort, concluding that perioperative PFMT may have selective benefits for women with preexisting pain syndromes, although it does not influence anatomical results or overall satisfaction.

Weidner et al. (2017) [47] also found no significant differences in pelvic floor impact (PFIQ), sexual function (PISQ-12), body image, or general health (SF-36) at 24 months between BPMT and standard care. Both groups showed sustained improvement from baseline, underscoring that surgery remains the main determinant of postoperative recovery.

Among the secondary literature, Espiño-Albela et al. (2022) [49] synthesized RCTs comparing perioperative PFMT versus standard care, finding consistent gains in muscle strength and reduced symptom distress (PFDI-20, PFIQ-7), but no effect on anatomical recurrence or POP-Q staging. The review supports integration of PFMT into peri- and postoperative pathways while calling for standardized, long-term protocols.

De Oliveira et al. (2024) [48] conducted a meta-analysis of PFMT after hysterectomy, reporting moderate-quality evidence of significant improvements in sexual function (+5 FSFI points) but uncertain effects on urinary symptoms and quality of life due to high heterogeneity. The review confirms PFMT as a safe, feasible, and potentially beneficial approach, though long-term structural effects remain unproven.

Shahid et al. (2025) [50], in a Cochrane review of 7 RCTs (1032 women), found no significant effect of perioperative PFMT on prolapse awareness, reoperation rates, or failure rates, and no clinically relevant changes in symptom or QoL scores.

Similarly, Zhang et al. (2016) [51] reported that perioperative PFMT did not significantly affect prolapse symptoms, quality of life, or POP-Q measures, reinforcing the notion that the strong surgical effect may mask any incremental rehabilitation benefit.

The International Urogynecology Consultation chapter by Bø et al. (2022) [52] reached the same conclusion, stating that no consistent evidence supports PFMT—before or after surgery—as a means to enhance surgical outcomes or prevent recurrence.

Finally, Basnet et al. (2020) [53], in a narrative review, summarized that structured perioperative PFMT programs demonstrate inconsistent efficacy and generally do not provide measurable advantages over surgery alone, despite being safe and well tolerated.

Two additional randomized controlled trials—Nyhus et al. (2020) [44] and Mathew et al. (2021) [42]—explored the effects of preoperative PFMT in women awaiting surgical repair for pelvic organ prolapse (POP).

Both studies implemented intensive, prolonged, and well-supervised programs lasting approximately 20–22 weeks, with high adherence rates (70–80%). Despite their methodological rigor and adequate statistical power, neither trial demonstrated significant differences between PFMT and standard care in postoperative outcomes, including pelvic floor muscle strength, prolapse symptom severity, or quality of life at six months.

These findings suggest that preoperative conditioning alone does not modify the postoperative recovery trajectory in women with advanced POP. Mechanical constraints imposed by prolapsed organs and the dominant corrective effect of surgery likely limit the potential impact of muscular training before anatomical restoration.

Taken together, these results complement the neutral findings of peri- and postoperative RCTs, underscoring that the timing and context of PFMT application are crucial determinants of its efficacy. The evidence indicates that PFMT achieves its full rehabilitative potential after surgical correction—when pelvic anatomy has been restored and neuromuscular recruitment, proprioception, and continence mechanisms can be effectively re-educated—rather than as a preoperative preventive measure.

### 4.2. Interpretive Reflection: Implications of Preoperative Evidence for Postoperative PFMT

The inclusion of these preoperative studies provides an important comparator for interpreting the peri- and postoperative literature.

Mathew et al. (2021) [42] found no added effect of preoperative PFMT on symptoms or quality of life related to urinary and colorectal-anal discomfort in women scheduled for POP surgery; postoperative symptomatic improvements were reached regardless of PFMT. Similarly, Nyhus et al. (2020) [44], showed no effect of preoperative PFMT on pelvic floor muscle contraction, POP symptoms, or anatomical prolapse after surgery. In all patients, POP symptoms improved at the 6-month follow-up, likely due to anatomical correction of the POP.

These consistent null findings indicate that the preoperative period is not the most effective therapeutic window for achieving lasting functional benefits—most likely because of the mechanical and neurophysiological distortion inherent to advanced prolapse.

By contrast, the postoperative phase, in which surgical correction restores pelvic anatomy, appears to provide a more favorable environment for neuromuscular re-education, recovery of voluntary control, and prevention of recurrence. In this sense, preoperative studies indirectly reinforce the overall conclusion of peri- and postoperative trials: PFMT is best conceptualized as a rehabilitative rather than a preventive intervention, whose efficacy depends on functional strengthening, individualized supervision, and sustained continuity after surgery.

## 5. Discussion

On the basis of the available evidence, randomized trials do not demonstrate a clear preventive effect of PFMT on postoperative anatomical outcomes or prolapse recurrence. However, when these findings are interpreted within current biomechanical and neuromuscular models of pelvic floor function, PFMT may be more appropriately conceptualized as a rehabilitative intervention aimed at functional recovery, neuromuscular re-education, and long-term maintenance of pelvic floor performance rather than as a strategy for short-term anatomical prevention. This perspective is primarily supported by narrative and theoretical contributions in the literature and should therefore be considered hypothesis-generating rather than directly evidence-proven by the studies included in the present review.

Several factors may explain the limited additional clinical impact of PFMT observed in available trials. Most rehabilitation protocols were short, typically lasting 6 to 12 weeks, which is likely insufficient to induce stable neuromuscular adaptations and long-term functional integration. Furthermore, many interventions lacked true individualization, using standardized exercise prescriptions that did not adequately address differences in baseline muscle function, symptom profiles, or patient-specific biomechanical characteristics. Adherence issues and variability in supervision intensity may have further attenuated treatment effects. These methodological and clinical limitations should be considered when interpreting the neutral findings of existing trials.

A key factor in understanding these results is the strong therapeutic effect of surgery itself. Surgical correction restores anatomy and pelvic support immediately, directly relieving symptoms such as heaviness, bulging, or the sensation of incomplete emptying. By repositioning the organs and re-establishing the appropriate tension of the fascial support system, surgery profoundly modifies the biomechanics and neurophysiology of the pelvic floor. Such a marked anatomical and functional impact can overshadow or mask any additional effects of PFMT, especially during the early postoperative months when outcomes are typically assessed. In almost all studies, both intervention and control groups showed substantial improvements in symptoms and quality of life, mainly attributable to the corrective effect of surgery. Under these conditions, it becomes difficult to identify incremental benefits of rehabilitation.

Another limiting factor concerns the structure of the PFMT protocols applied. Most perioperative programs were short-term—generally lasting between 6 and 12 weeks—and focused on isolated muscle activation rather than on progressive, functional, and integrated approaches. True neuromuscular adaptation, however, requires time, repetition, and progressive loading. Short or non-individualized interventions rarely support stable motor learning, movement automatization, and integration into daily motor patterns. Therefore, longer, progressive, and individualized rehabilitation protocols may be required to achieve stable muscular recovery and to translate physiological gains into clinically meaningful outcomes. Exercises should nevertheless be prescribed according to the same principles and modalities that have proven effective in the conservative management of POP, as described in the protocols by Brækken et al. [5] and subsequent systematic reviews, which precisely define the parameters of intensity, frequency, and duration of pelvic floor muscle training. In line with the recommendations of the American College of Sports Medicine (ACSM) [55], training should be sufficiently intense, repetitive, and personalized to optimize the recovery of strength, power, and muscular endurance, thereby ensuring functional consolidation and preventing recurrences or de novo dysfunctions.

Several studies support the efficacy of PFMT when performed with adequate intensity and continuity: only a truly strong pelvic floor can counteract prolapse recurrence, as demonstrated by its effect on reducing low-grade prolapse and treating stress and urgency urinary incontinence. In all these contexts, clinical benefits emerge only when the exercise is correctly executed, with load progression and professional supervision, confirming that the quality of training represents the essential basis for functional recovery and long-term stability of surgical and rehabilitative outcomes.

Moreover, the outcome measures used—often symptom questionnaires or anatomical assessments—may be too coarse to detect subtle improvements in proprioception, endurance, or coordination that PFMT can induce.

From a biomechanical perspective, the type of exercise is also of fundamental importance: coordination among the pelvic floor, abdominal wall, and respiratory dynamics is crucial after POP surgery. In some studies, women performing PFMT in combination with abdominal exercises reported sensations of tension, heaviness, or downward pressure—signs that occur when intra-abdominal pressure is poorly controlled during training [31]. This highlights a crucial principle: re-educating the musculature, rather than simply treating symptoms, is essential for long-term pelvic stability. Effective rehabilitation should therefore include respiratory education, pressure control, and coordinated activation of pelvic, abdominal, and postural musculature. Such integrated re-education optimizes function and may help prevent recurrences or the onset of new dysfunctions over time. The use of hypopressive techniques, to date, has shown no proven efficacy [56,57].

To achieve these objectives, PFMT should be incorporated into personalized, progressive, and multidisciplinary rehabilitation pathways rather than proposed as a short-term perioperative intervention. The impact of surgery on prolapse makes it unlikely to observe measurable PFMT effects in the early postoperative phase; however, once healing is complete and mechanical correction stabilized, training becomes crucial to transform anatomical repair into functional competence. In this phase, muscle re-education promotes recovery of control, awareness, and body confidence, consolidating surgical outcomes and integrating them into daily motor patterns.

From a physiological standpoint, the post-surgical stabilization phase represents a particularly favorable moment for neuromuscular recovery. Once normal anatomy is restored, the pelvic floor can be retrained under optimal conditions, leading to improvements in proprioception, motor control, and continence reflexes. In this sense, PFMT should not be regarded as an accessory or merely a preventive intervention, but rather as a rehabilitative process that consolidates surgical repair by restoring coordinated neuromuscular function within the reconstructed anatomy. Moreover, targeted exercises may exert a selective effect on postoperative pain modulation (see literature on exercise and post-surgical pain mechanisms), underscoring the importance of individualized programs tailored to the patient’s preoperative symptom profile.

The rationale for postoperative PFMT extends beyond muscle strengthening to encompass recovery of neuromuscular integration and tissue healing. Pelvic surgery may temporarily impair the structural and functional integrity of the pelvic floor muscles and connective tissues that support the bladder and organs [48]. Training interventions improve coordination, endurance, and vascularization of the pelvic floor, thereby promoting optimal muscle recruitment and enhancing pelvic support [35,38,43]. These mechanisms contribute not only to the prevention of recurrence but also to the recovery of continence, defecatory efficiency, and sexual function.

Conceptually, this rehabilitative approach parallels the Enhanced Recovery After Surgery (ERAS) paradigm, which emphasizes early mobilization, multimodal rehabilitation, and optimization of functional recovery [26]. ERAS protocols primarily focus on optimizing the immediate postoperative phase through measures such as optimized fluid management, multimodal analgesia, early mobilization, reduction in complications, and shortening of hospital stay. In this context, the principles of ERAS provide a conceptual background supporting early functional activation and patient engagement. However, the structured rehabilitation programs discussed in the present review extend beyond the ERAS time window and should be regarded as complementary long-term functional strategies rather than direct components of ERAS pathways. Integrating PFMT within ERAS-oriented pathways may therefore facilitate more complete healing, minimize pain and stiffness, and accelerate the restoration of pelvic function and quality of life after prolapse repair.

Interventions specifically addressing patients with pre-existing pain or hypertonia may improve comfort, adherence, and the overall recovery trajectory.

Clinically, PFMT is a safe, feasible, and well-tolerated intervention. Although adverse events were not a primary outcome in the included studies, available evidence consistently indicates that PFMT is a safe intervention with a very low incidence of complications. In the most recent Cochrane review, 9 of 63 trials reported adverse events, with 66 events among 1083 participants (6%). Almost all were minor and transient, consisting mainly of vaginal discharge, spotting, or local discomfort. Importantly, these events were largely associated with intravaginal or intrarectal training devices rather than with PFMT itself, and no serious adverse effects were reported [58]. High-quality evidence consistently demonstrates that PFMT has an excellent safety and tolerability profile. Adverse effects are rare, generally mild and transient, and consist mainly of occasional discomfort or pain, with no serious complications reported [59]. More recent randomized trials further confirm these findings. No adverse events were observed during an eight-week supervised multimodal PFMT program, with perfect attendance (100%) and high participant satisfaction (84.6%) [60]. Similarly, in a 12-week PFMT randomized trial including 126 women, high adherence, significant functional and quality-of-life improvements, and no clinically relevant complications attributable to PFMT were reported [61].

Overall, PFMT should be considered an essential complement to prolapse surgery and implemented within sufficiently long, guided, and individualized programs tailored to each patient’s functional profile. A structured, continuous rehabilitation plan allows the long-term maintenance of gains and enhances the contribution of surgery to overall quality of life. To optimize long-term functional recovery, PFMT should be integrated into structured postoperative care pathways rather than delivered as an isolated intervention. Integration should include early postoperative assessment of pelvic floor function, individualized rehabilitation planning based on symptom profile and neuromuscular deficits, progressive supervised training phases, and scheduled long-term follow-up to support maintenance and adherence. Multidisciplinary collaboration among surgeons, physiotherapists, nurses, and primary care providers is essential to ensure continuity of care and timely adjustment of treatment strategies. Embedding PFMT within standardized postoperative pathways enables functional rehabilitation to directly complement anatomical repair, addressing persistent or de novo dysfunctions and improving durable quality-of-life outcomes.

Adherence to treatment also represents a significant barrier. In the postoperative phase, some patients fear that contractions might interfere with healing or cause pain, while others perceive exercise as unnecessary after surgical correction. These behavioral and motivational barriers compromise training continuity and attenuate its effects. However, when educational support and professional supervision are maintained over time, adherence rates and clinical outcomes improve significantly, confirming that the quality, duration, and personalization of the therapeutic pathway are as decisive as the exercise protocol itself. Improving adherence to PFMT in clinical practice requires structured, theory-driven, and patient-centered strategies. According to the International Continence Society State-of-the-Science report [62], key modifiable determinants of adherence include self-efficacy, positive intention to adhere, perceived benefits of the exercises, attitudes toward treatment, and integration of PFMT into daily activities. Effective strategies identified in the literature include individualized exercise prescription with goal setting, regular supervised follow-up, enthusiastic therapist engagement, structured treatment protocols, and the use of behavioral change models. Additional supportive tools, such as exercise diaries, audio prompts, reminder systems, and digital monitoring, may further enhance long-term adherence. These elements appear essential for translating neuromuscular improvements into durable functional outcomes.

From an applied standpoint, current evidence supports directing postoperative rehabilitation toward selected patients with residual or de novo dysfunctions, rather than universally prescribing it to all women undergoing POP surgery. A selective approach allows for optimization of resources and maximization of therapeutic impact. At the same time, future studies should accurately report PFMT parameters—intensity, frequency, supervision, and progression—since most studies failed to provide essential details, thereby limiting reproducibility and interpretation. Defining standardized, transparent, and replicable rehabilitation models is a key step in clarifying the actual clinical value of PFMT after POP surgery.

Based on the available randomized evidence, there is currently no high-level evidence that multimodal pelvic floor muscle training (MPFT) after prolapse surgery provides additional global benefits over surgery alone across different prolapse types. Nevertheless, from a clinical and pathophysiological perspective, individualized postoperative rehabilitation remains a reasonable and potentially valuable approach for managing persistent or de novo pelvic floor dysfunctions, including overactive bladder, stress urinary incontinence, pelvic pain, and dyspareunia. This strategy should be considered a targeted functional intervention aimed at symptom relief and quality-of-life improvement rather than a universally preventive or outcome-modifying treatment.

## Figures and Tables

**Table 1:** Summary of functional and anatomical outcomes reported after POP surgery.

Outcome	Reported Rate Range	Main References	Follow-Up Duration
Persistence of prolapse awareness	17–23%	–	–
Stress urinary incontinence (SUI)	8–15%	–	–
De novo SUI	4–50%	Tran 2017 [22]; Yeung 2024 [14]	–
Urgency/Overactive bladder symptoms	5–30%	Tran 2017 [22]; Maher 2023 [16]; Deffieux 2024 [13]; Yeung 2024 [14]	–
Dyspareunia	5–17%	Maher 2016 [12]	–
Obstructed defecation/Emptying disorders	7–60%	Tran 2017 [22]; Deffieux 2024 [13]	–
De novo dyspareunia	~7%	Mortier 2020 [21]; Maher 2023 [16]; Deffieux 2024 [13]; Yeung 2024 [14]	24 months → 5 years
Sexual dysfunction	6–14%	Wihersaari 2024 [23]; Antosh 2021 [24]	1 year → 5 years
Obstructed defecation	2–37%	Sung 2012 [18]; Deffieux 2024 [13]	12 months
Fecal incontinence	2–15%	Ballard 2015 [19]; Maher 2023 [16]	–
Prolapse recurrence requiring re-surgery	up to 20%	Chen 2023 [9]; Zhang 2020 [10]	–
Symptomatic prolapse recurrence	1.4–17.4%	Zhang 2020 [10]; Schulten 2022 [11]	–
De novo pelvic pain	2.5–17%	Singh 2022 [20]; Vancaillie 2018 [21]; Maher 2023 [16]; Deffieux 2024 [13]	4 months → 8 years

**Table 2:** Detailed extraction and critical appraisal of randomized controlled trials (RCTs). Overall results: Small sample, short follow-up, limited power to detect differences. Abbreviations: RCT: randomized controlled trial; Exp: experimental; Cont: control; SUI: stress urinary incontinence; POP: pelvic organ prolapse; SSLF: sacrospinous ligament fixation; ULS: uterosacral ligament suspension; BPMT: behavioural pelvic floor muscle training; UDI: Urinary symptoms Inventory; POPDI: prolapse symptoms; PFMT: pelvic floor muscle training; P-QOL: Prolapse quality of life; PBU: pressure biofeedback unit; PFPT: pelvic floor physical therapy; WHOQOL-BREF: World Health organization quality of life-brief version; PFDI: Pelvic Floor Distress Inventory—20 items; PFIQ-7: Pelvic Floor Impact Questionnaire—short form 7; FSFI: Female Sexual Function Index; VAS: visual analogue scale; PISQ-12: Pelvic Organ Prolapse/Urinary Incontinence Sexual Questionnaire; IIQ: incontinence impact questionnaire; CRADI: colorectal-anal distress inventory; POPDI: pelvic organ prolapse distress inventory; PFDI: pelvic floor distress inventory; PGI-I: patient global impression of improvement; LA: lifestyle advice; SF-36: short form-36; POP-IQ: pelvic organ prolapse impact questionnaire; CRAIQ: colorectal-anal impact questionnaire; UIQ: urinary impact questionnaire; POP-SS: pelvic organ prolapse symptoms score; ICIQ-UI SF: International Consultation on Incontinence Questionnaire-Urinary Incontinence Short Form; ICIQ-BS; SF: International Consultation on Incontinence Questionnaire-Bowel symptoms—Short Form.

Author (Year, Country)	Study Design	Population	POP Type/Surgical Technique	Intervention/Follow Up	Primary Outcome	Urinary/Bowel/Sexual/Pain Symptoms	Objective vs. Subjective Outcomes	Main Conclusions	Declared Limitations	Drop-Out/Adherence	PEDro Score (0–10); Risk of Bias Tool Score with Main Critical Points
Barber et al., 2014 (USA) [33]	Multicenter RCT	374 women with stage II–IV apical vaginal prolapse and SUI	Vaginal prolapse surgery with concomitant mid-urethral sling; randomized to SSLF, n = 186) vs. ULS, n = 188	EXP: BPMT-1 pre-op + 4 post-op sessions up to 12 weeks Cont: usual cares Follow up: 24 months	Surgical success at follow up; BPMT: UDI at 6 mo, POPDI + anatomic failure	UDI, POPDI, bowel symptoms, pelvic floor strength (Brink score), adverse events	Both objective (POP-Q, retreatment, adverse events) and subjective (PFDI/UDI/POPDI)	No significant differences between SSLF and ULS in surgical success. BPMT did not improve urinary or prolapse outcome at 6–24 mo.	Lower success compared to case series, not powered for interaction between surgery × BPMT. Findings not generalizable to mesh or abdominal repairs.	Drop-out: 14–18% across groups; BPMT adherence: 93% at 6 months, 81% at 24 months	8/10; Some concerns Criticalities Deviations: some concerns (difficult to blind surgeons, perioperative care variations). Missing data: some concerns (multiple imputation; ~15% dropout at 24 months).
Borello-France et al., 2023 (USA) [34]	RCT	186 women scheduled for POP surgery	Various vaginal prolapse repairs (anterior, posterior, apical)	Exp: Perioperative PFMT program (behavioral therapy + home exercises, supervised sessions) Cont: usual cares Follow up: 24 months	Adherence to PFMT	Not evaluated	Objective: adherence logs; Subjective: self-report adherence	Adherence was moderate; home exercise adherence declined over time. Study established that adherence of perioperative training did not influence 24-month outcome	Small sample, focus limited to feasibility/adherence, not generalizable to all surgical approaches.	Drop-out ~15%; adherence moderate (<70% by end of follow-up)	06/10; Some concerns Criticalities: Deviations: some concerns (unblinded participants/therapists).
Brandt & Janse van Vuuren, 2022 (South Africa) [35]	Double-blind 3-arm RCT	81 women undergoing pelvic floor reconstructive surgery (stage II–III predominant); single surgeon across two hospitals	Mixed reconstructive procedures (e.g., sacrospinous fixation, rectocele plication with perineal body repair, sacrocolpopexy; anterior/posterior repairs)	Group 1: PFMT (individualized progression, biofeedback/US/EMG-assisted); Group 2: Abdominal training + PFMT; Group 3: standard care only Follow up: 6 months	P-QOL and pelvic floor/abdominal function (PERFECT scheme, EMG, perineal US, PBU, Sahrmann)	Urinary frequency ↑ at 3 months group 2 vs. 3; bulging/discomfort ↑ at 6 months groups 2 vs. 3; pain	Objective: POP-Q, perineal ultrasound, EMG, PBU; Subjective: P-QOL, symptom scales, pain	No between-group benefit on QoL or POP symptoms at 6 months. PFMT improved PFM function (power, fast contractions, endurance. Abdominal + PFMT improved abdominal measures but increased discomfort.	Short follow-up, missing data due to transport barriers; resource-limited setting; heterogeneity of procedures.	Attrition at 6 mo: 2 (PFMT), 2 (Abdominal + PFMT), 3 (Control). Exercise compliance > 60% at 3 months; >55% at 6 months.	06/10; Some concerns Criticalities Deviations: some concerns (therapist/participant blinding imperfect). Missing data: some concerns (attrition at 6 mo).
Dawson et. al., 2018 (USA) [36]	RCT (Conference Abstract)	n = 46 women after vaginal reconstructive surgery for POP	Vaginal reconstructive surgery	Exp: 6-week postoperative PFPT protocol Cont: standard post-operative care Follow up: 6 w + 12 w	WHOQOL-BREF, PFDI-20, PFIQ-7, FSFI, VAS (pain/sexual pain), POP-Q	FSFI, VAS	All subjective measures	Significant improvement in pain and QOL in both groups, no difference between PFPT and standard care	Abstract only; small sample; short follow-up; no detailed randomization/blinding; high attrition	Not reported	N/A
Duarte et al., 2020 (Brazil) [37]	Parallel-group RCT	96 women with POP stage II–IV and bulge symptoms (age 35–80)	Vaginal repairs: anterior, apical and/or posterior compartment surgery Follow up: 3 (Day 40 and Day 90)		PFDI-20 total (0–300); subscales POPDI-6, CRADI-8, UDI-6	PFDI subscales; sexual function (PISQ-12)	Objective: vaginal manometry (Peritron) peak/mean/endurance; Subjective: PFDI-20, PFIQ-7, PISQ-12, PGI-I	Perioperative PFMT added to POP surgery produced no clinically relevant improvement in POP symptoms, PFM strength, QoL, or sexual function at 40–90 days.	Short intervention and follow-up may have limited detection of PFMT effects; despite high adherence, PFMT did not improve PFM strength.	Not reported	Moderate quality RCT Criticalities Deviations: some concerns (participants/therapists unblinded). Missing data: low to some concerns (minimal attrition).
Frawley et al., 2010 (Australia) [38]	Assessor-blinded RCT	51 women undergoing vaginal or laparoscopic-assisted vaginal POP repair and/or hysterectomy	Mixed POP repairs and/or hysterectomy across six private hospitals; no objective POP-Q assessment Follow up: 12 (pre-op, 3, 6, 12 months)		UDI-19 and IIQ-7	UDI Irritative, Stress), POP obstructive; Wexner; Constipation.	Objective: 3-day bladder diary; 48 h pad test; vaginal manometry. Subjective: UDI, IIQ, AQoL; digital muscle test		Small sample size; heterogeneity of surgical procedures; low and uneven recruitment; potential contamination because “usual care” often included advice and PFMT; physiotherapists and participants were not blinded; adjunctive therapies used only in some TG participants; lack of POP-specific assessment	Very low dropout (≈4% in both groups). TG adherence high during supervised phase (89% session attendance; 71% diary return; 89% adherence to exercise dosage), but declined during unsupervised phase.	Moderate quality RCT Criticalities Randomization: some concerns (baseline imbalance; unclear concealment). Deviations: some concerns (unblinded; contamination).
Jarvis et al., 2005 (Australia) [39]	RCT	60 women scheduled for POP and/or incontinence surgery; 30 intervention, 30 control	Mixed POP and/or incontinence procedures across tertiary hospital; heterogeneous surgical techniques	Exp: Individualized PFMT (4 sets/day), ‘Knack’, bladder/bowel training; reinforced immediately post-op and at 6 weeks Cont: standard perioperative care Follow up:3 months	Urinary symptoms (QoL questionnaire), paper towel test, PFM manometry, frequency-volume diary	Significant improvements in urinary symptoms, QoL, diurnal frequency, and maximum PFM squeeze in intervention group; bowel/sexual/pain not assessed	Objective: paper towel test, manometry, Oxford scale; Subjective: urinary symptoms and QoL questionnaires, voiding diary	Perioperative physiotherapy improved urinary symptoms, pelvic floor strength, and QoL compared with surgery alone	Small sample size, short follow-up, surgical heterogeneity, limited generalizability	≈7% attrition (3 intervention, 1 control cancelled surgery); adherence reinforced peri- and postoperatively	06/10; Some concerns Criticalities Deviations: some concerns (no blinding of participants/therapist); Missing data: some concerns (attrition, cancellations);
Jelovsek et al., 2018 (USA) [40]	Multicenter RCT	374 women with stage II–IV apical POP and SUI; 285 enrolled in extended trial	Vaginal apical repairs: Uterosacral ligament suspension (ULS) vs. Sacrospinous ligament fixation (SSLF), with concomitant midurethral sling in all	Exp: Perioperative behavioral therapy + PFMT (BPMT): 1 pre-op + 5 post-op sessions, individualized exercises up to 45–60/day Cont: usual perioperative care Follow up: 60 months	Time to surgical failure (POP-Q failure, bulge symptoms/retreatment); time to anatomic failure; POPDI	UDI, CRADI, POPDI; sexual and pain outcomes assessed secondarily	Objective: POP-Q, retreatment, adverse events; Subjective: PFDI subscales (POPDI, UDI, CRADI), PGI-I	No significant differences between ULS vs. SSLF or BPMT vs. usual care at 5 years. Failure rates high (ULS 61.5%, SSLF 70.3%), but QoL and symptom improvements sustained	High surgical and anatomic failure rates at 5 years; selective dropout may bias results; stringent composite definitions may overestimate ‘failure’; behavioral intervention may have been too short-dose	374 randomized, 285 entered extended trial, 244 (86%) completed 5-year follow-up; adherence to BPMT moderate	08/10; Some concerns Criticalities Deviations: some concerns (unblinded participants/therapists). Missing data: some concerns (selective follow-up, attrition).
Liang et al., 2019 (China) [41]	RCT	97 randomized (49 LA + PFMT, 48 LA); 90 completed (47 and 43 analysed)	POP stage III–IV; surgical approach not specified beyond standard vaginal repairs	Exp: PFMT + lifestyle advice: 4 sessions; daily PFMT (100–150 contractions/day); nurse-supervised during hospitalization Cont: LA Follow up: 2 months	PFDI-20 and subscales (UDI-6, CRADI-8, POPDI-6)	Significant improvement in UDI-6 in PFMT + LA group at 42 and 60 days; no between-group differences for POPDI-6 and CRADI-8; both groups improved over time	Subjective only: PFDI-20 (Chinese version)	PFMT after POP surgery improved urinary symptoms beyond LA alone; overall POP and bowel symptoms improved similarly in both groups	Short follow-up (2 months), no exercise logs to verify adherence, single-center, surgical details limited; only one subjective questionnaire used	Low attrition (4 dropouts total); adherence uncertain (self-reported, no exercise logs)	06/10; Some concern Criticalities Deviations: some concerns (participants/therapists not blinded); Measurement: some concerns (only subjective outcomes, no POP-Q)
Mathew et al., 2021 (Norway) [42]	RCT	159 randomized (81 intervention, 78 control); 151 completed (75 vs. 76)	Mixed POP stage ≥ II; surgical procedures included anterior/posterior colporrhaphy, vaginal hysterectomy, sacrospinous fixation, laparoscopic sacrocolpopexy, sacrohysteropexy	Exp: Preoperative PFMT: daily intensive program (8–12 maximal contractions, 6–8 s hold, 3×/day) for ~22 weeks; supervised by physiotherapists at baseline, 2 and 6 weeks; adherence diaries Cont: waiting list Follow up:6 months	UDI-6, CRADI-8, UIQ, CRAIQ	No significant differences between groups in UDI-6, CRADI-8, UIQ, CRAIQ at surgery or 6 months; all groups improved significantly after surgery; adherence 80% ≥ 70% training	Subjective only: validated Norwegian PFDI-20 and PFIQ-7 subscales	Preoperative PFMT did not add benefit beyond surgery; surgery alone improved urinary and colorectal-anal distress and QoL	No assessor blinding for clinical exam; possible PFMT contamination in controls not excluded; short follow-up; no objective POP-Q effect reported here	151/159 completed (95%); adherence to PFMT high (80% ≥ 70%)	07/10 Some concerns Criticalities Deviations: some concerns (not blinded participants/therapists); Measurement: some concerns (no blinding, subjective outcomes only).
McClurg et al., 2014 (UK) [43]	Multicenter RCT (feasibility study)	57 women undergoing POP surgery; randomized 28 intervention, 29 control; mean age ≈ 60	Stage II–III POP, mostly anterior; vaginal prolapse repairs	Exp: Perioperative PFMT: 1 pre-op + 6 structured post-op sessions; LA; adjuncts allowed (biofeedback, e-stim); supervised by physiotherapists Cont: usual cares Follow up: 12 months	POP-SS; secondary: POP-Q, PFM assessment, ICIQ-UI SF, ICIQ-BS, PISQ-12, SF-12	Improvements in POP-SS, UI, bowel, and QoL in both groups; greater symptom reduction at 12 months in PFMT group; bowel and sexual function changes limited	Objective: POP-Q, PFM manometry, Oxford, PERFECT; Subjective: POP-SS, ICIQ, PISQ-12, SF-12	PFMT adjunct may reduce prolapse symptoms after surgery, but pilot nature and limited 12-month data mean results are not definitive; feasibility for larger RCT confirmed	Recruitment slow at some centers; 12-month data mainly from 1 site; high attrition at 12 months; pilot underpowered	Retention: 82–86% at 6 mo, ~50% at 12 mo; adherence high at site that recruited to target; home diary completion poor	06/10; Some concerns Criticalities Deviations: some concerns (unblinded participants/therapists); missing data: high (attrition at 12 months)
Nyhus et al., 2020 (Norway) [44]	RCT	159 randomized women with symptomatic POP stage ≥ II scheduled for surgery (81 intervention, 78 control);	Mixed surgical approaches (anterior/posterior colporrhaphy, vaginal hysterectomy, sacrospinous fixation, sacrohysteropexy, sacrocolpopexy; native tissue ± mesh)	Exp: Preoperative PFMT: daily training (8–12 contractions, 6–8 s, 3×/day) for ~3 months; supervised by physiotherapist at 2 and 6 weeks; Cont: no intervention Follow up: 6 months	PF muscle contraction (MOS, manometry, EMG, ultrasound hiatal APD), POP symptoms (vaginal bulge VAS), anatomical prolapse (POP-Q, US)	No differences between groups for PFM contraction, POP symptoms, or anatomic prolapse at 6 months. All patients improved after surgery (PFM contraction, POP symptoms, anatomic descent)	Objective: MOS, manometry, EMG, transperineal US, POP-Q; Subjective: bulge VAS	Preoperative PFMT did not improve surgical outcomes; improvements due to surgery alone	No assessor blinding post-op for palpation; heterogeneous surgical procedures; possible contamination (controls instructed in correct contraction at baseline); short follow-up	151/159 completed (95%); adherence to PFMT high (80% ≥ 70% adherence)	07/10 Some concerns Criticalities Deviations: some concerns (not blinded); Measurement: some concerns (no blinding of palpation, partial missing data).
Pauls et al., 2014 (USA) [45]	Single-center RCT	49 women undergoing native tissue vaginal reconstructive surgery for POP (24 PFPT, 25 control)	Vaginal hysterectomy, anterior/posterior repair, enterocele repair, vault suspension; some with concomitant sling	Exp: PFPT: 1 pre-op + biweekly sessions until 12 weeks post-op (6 total); supervised exercises, EMG-based training, bladder/bowel advice Cont: usual cares Follow up: 6 months	WHOQOL-BREF (primary); PFDI-20, PFIQ-7, SF-12, PISQ-12, FSFI; Oxford scale; voiding diary; POP-Q; intravaginal EMG	Improvements in all domains of QoL and function at 12 and 24 weeks in both groups; EMG gains at 12 weeks in PFPT group disappeared by 24 weeks; sexual function (FSFI, PISQ-12) improved in both groups by 24 weeks; bladder symptoms improved (UDI-6, IIQ-7)	Objective: EMG, Oxford, POP-Q; Subjective: WHOQOL-BREF, PFDI-20, PFIQ-7, SF-12, PISQ-12, FSFI, diaries	Vaginal reconstructive surgery improved QoL, bladder and sexual function regardless of PFPT; no added benefit of PFPT at 6 months	Small sample, single-center, short follow-up, limited power to detect differences; EMG benefit transient	49/57 completed (86%); adherence to PFPT good during first 12 weeks	06/10 Some concerns Criticalities Randomization: low risk; Deviations: some concerns (not blinded); Missing data: low risk; Measurement: some concerns (EMG reproducibility, no blinded assessors); Reporting: low risk
Wang et al., 2023 (USA) [46]	Secondary analysis of multicenter RCT	368 women (109 with preoperative pelvic pain, 259 without); mean age ~57	Vaginal apical repair: USLS vs. SSLF, both with concomitant midurethral sling	Exp: Perioperative PFMT vs. usual care, as in OPTIMAL study Cont: Usual care Follow up: 24 months	Change in pain scores across 24 months; PFDI, PFIQ, PGI-I; surgical success at 24 months	Women with preoperative pain had greater improvements in pain, UDI, POPDI, CRADI, and QoL vs. those without pain; in SSLF subgroup, PMT further reduced pain persistent pain at 24 mo in 16% with pre-op pain	Objective: surgical success (POP-Q, retreatment, bulge symptoms); Subjective: pain scale, PFDI, PFIQ, PGI-I	Vaginal reconstructive surgery improved pain and pelvic floor symptoms regardless of pre-op pain; PMT may benefit women with pain undergoing SSLF	Secondary analysis; pain not prespecified as primary endpoint; pain diagnoses not classified; no validated chronic pain scale; residual confounding	Derived from OPTIMAL: 368/374 with pain data included; adherence to PMT moderate	N/A; Some concerns Criticalities Deviations: some concerns (non-blinded PMT); Measurement: some concerns (pain scale validity, no stratified chronic pain dx)
Weidner et al., 2017 (USA) [47]	Secondary analysis of RCT (OPTIMAL trial)	374 women with POP stage II–IV and SUI; mean age 57; randomized to BPMT (n = 186) vs. usual care (n = 188)	Vaginal apical suspension: ULS vs. SSLF, both with concomitant midurethral sling	Exp: Perioperative BPMT: 5 visits (1 pre-op, 4 post-op), supervised by certified nurses/PTs, included PFMT and bladder/bowel strategies Cont: usual care Follow up: 24 months	Change in QoL: PFIQ short form (POP-IQ, UIQ, CRAIQ), SF-36, PISQ-12, body image, PGI-I, Brink score	No significant differences between BPMT and usual care at 24 mo in QoL, body image, sexual function, PGI-I, or Brink scores. Surgery alone improved all outcomes significantly	Objective: Brink score POP-Q; Subjective: PFIQ, SF-36, PISQ-12, PGI-I, body image	Perioperative BPMT did not add benefit to surgery for POP + SUI; surgery alone improved QoL and sexual function	Clinicians varied in expertise; findings may not generalize beyond transvaginal POP + SUI repairs with sling; not powered for subgroup analysis	Retention: 74% BPMT, 78% UC at 24 mo; adherence self-reported high (81% at 24 mo)	Not applicable (secondary analysis)

**Table 3:** Critical appraisal of systematic reviews included in the scoping review on rehabilitation after pelvic organ prolapse (POP) surgery. Overall results: 4 systematic reviews; conclusion: Low; Critical findings. Abbreviations: PFMT: pelvic floor muscle training; PFM: pelvic floor muscle; QoL: quality of life; RCT: randomized controlled trial; POP: pelvic organ prolapse; BFB: biofeedback; UDI: Urinary symptoms Inventory; CRADI: colorectal-anal distress inventory; PFDI: Pelvic Floor Distress Inventory; UTI: urinary tract infection.

Author (Year, Country)	Objective	Databases and Search	Studies and Participants	Main Findings	AMSTAR 2 Appraisal	Overall Confidence Rating	Critical Appraisal Notes	Funding/Conflicts Reported
de Oliveira et al., 2024 (Brazil) [48]	Evaluate the effects of PFMT on urinary symptoms, vaginal prolapse, sexual function, PFM strength, and QoL in women after hysterectomy	CINAHL, Cochrane, Embase, MEDLINE Ovid, PEDro, PubMed; Feb–Mar 2022, updated Oct 2023; PROSPERO CRD42020198000	6 RCTs (8 reports), 776 women	Sexual function improved (+5 FSFI points, clinically relevant); possible benefit for PFM strength, urinary symptoms, QoL; no effect on prolapse; long-term sustainability uncertain	Protocol preregistered; comprehensive search; duplicate selection; no full excluded list; RoB via PEDro only; no funding info; no publication bias assessment	Low (critical flaws in RoB, funding, excluded studies, publication bias)	Well-conducted with PRISMA & GRADE; but reliance on PEDro, incomplete exclusions list, and no publication bias assessment lower confidence	Funding of included trials not reported; conflicts of interest declared by review authors
Espiño-Albela et al., 2022 (Spain) [49]	Evaluate the effectiveness of PFMT in women with POP treated conservatively vs. surgically (RCTs)	PubMed, Scopus, CINAHL, Cochrane, PEDro; Apr–Oct 2021; PRISMA compliant; not PROSPERO registered	18 RCTs, >2300 women (1034 related to PFMT + BFB + Lifestyle advice)	PFMT improves pelvic, urinary, and bowel symptoms, QoL, and PFM strength; no consistent benefit for POP stage or sexual function; no added effect when combined with surgery	No preregistration; comprehensive search; duplicate selection; no excluded study list; RoB via PEDro only; no funding info; narrative synthesis only; no publication bias assessment	Critically low (multiple critical flaws: no protocol, no funding report, no excluded studies, no meta-analysis, limited RoB)	Comprehensive overview, clinically useful, but low methodological rigor; findings consistent with higher-quality reviews, but confidence downgraded	Funding of included trials not reported; conflicts not detailed
Shahid et al., 2025 (Cochrane Collaboration; multinational) [50]	Evaluate the safety and effectiveness of perioperative interventions in women undergoing POP surgery	Cochrane Incontinence Group Specialized Register (CENTRAL, MEDLINE, CINAHL), ClinicalTrials.gov, WHO ICTRP, handsearching; last search Apr 2024; protocol registered	49 RCTs, 5657 women, 19 intervention categories (1032 related to PFMT)	PFMT perioperatively (7 RCTs, 1032 women): little/no effect on prolapse awareness, repeat surgery, objective failure, PFDI/UDI/CRADI; prolonged catheterization > 24 h ↑ UTI risk (OR 9.25); other interventions (bowel prep, antiseptics, estrogen, cranberry, vaginal packing, activity restriction) showed no clear benefit	Protocol preregistered; exhaustive search; duplicate selection; excluded study list provided; RoB by Cochrane tool; funding partially reported; meta-analysis robust; publication bias assessment limited	High (Cochrane rigor; only minor limitations, most evidence low-certainty due to small trials/heterogeneity)	Methodologically robust with GRADE; broad scope; certainty mostly low–moderate; strongest signal: prolonged catheterization ↑ UTI risk; PFMT adds little to POP surgery outcomes	Funding of included trials partially reported; review authors declared conflicts
Zhang et al., 2016 (China) [51]	Determine whether perioperative PFMT improves outcomes of POP surgery	PubMed, Embase, Cochrane Library, Web of Science; inception–Jun 2014; Google Scholar; no protocol registration	5 RCTs, 591 women (TG 292 vs. CG 299)	No significant added benefit of PFMT for prolapse symptoms, QoL, or POP stage; some transient gains in PFM function and urinary outcomes in small RCTs; adherence good; no adverse events	No protocol; broad search; duplicate screening; no excluded studies list; RoB assessed with Cochrane tool but reporting limited; no funding info; no meta-analysis	Low (critical flaws: no preregistration, no funding, no excluded studies, qualitative only)	Early systematic attempt; concluded insufficient evidence for perioperative PFMT; strengths: broad search and RoB; weaknesses: heterogeneity, small underpowered RCTs, no quantitative synthesis	Funding of included trials not reported; conflicts not declared

**Table 4:** Critical appraisal of narrative reviews included in the scoping review on rehabilitation after pelvic organ prolapse (POP) surgery. Overall results: two narrative reviews positively evaluate the role of physiotherapy associated with POP surgery. Abbreviations: PFMT: pelvic floor muscle training; POP: pelvic organ prolapse; RCTs: randomized controlled trials.

	Journal/Type of Review	Objective/Focus	Sources/Search Description	Main Findings/Key Concepts	Declared Limitations	Critical Appraisal (SANRA Domains)	Overall Quality
Basnet P., Yong L., Sujanshe J., et al. (2020) [52]	Int Urogynecol J—Narrative review	To summarize current evidence and clinical perspectives on the role of physiotherapy and PFMT in women undergoing POP surgery, including timing and rationale.	Non-systematic review of published literature; databases not specified; reference-based discussion.	PFMT before and after surgery may optimize outcomes, reduce recurrence, and improve urinary, bowel, and sexual symptoms; early postoperative rehabilitation is advocated.	Non-systematic design; lack of detailed search and quality assessment; conclusions partly opinion-based.	Justification 2; Aims 2; Literature search 1; Referencing 2; Scientific reasoning 2; Presentation 2; Total = 11/12.	High-quality narrative review—well-structured, coherent reasoning despite absence of systematic search.
Bø K., Nyhus B.E., Hilde G. (2022) [53]	Int J Urogynecol-Narrative review	To review current knowledge on PFMT before and after POP surgery, summarizing evidence, mechanisms, and clinical recommendations.	Narrative synthesis of contemporary evidence from RCTs, systematic reviews, and clinical studies; databases not specified.	Strong rationale for PFMT before and after POP surgery. Preoperative training may improve awareness and strength; postoperative PFMT enhances recovery and continence. Evidence supports integration of physiotherapy into surgical care pathways.	Narrative methodology without systematic search strategy; limited quantitative synthesis; expert interpretation-based.	Justification 2; Aims 2; Literature search 1; Referencing 2; Scientific reasoning 2; Presentation 2; Total = 11/12.	High-quality narrative review—authoritative synthesis integrating clinical and research perspectives.

**Table 5:** Critical appraisal of expert opinion and consensus papers included in the scoping review on rehabilitation after pelvic organ prolapse (POP) surgery. Overall results: Only one clinical opinion/expert commentary from 2013 supports early physiotherapy and PFMT before and after pelvic organ prolapse (POP) surgery. Two small randomized controlled trials (RCTs) were identified, differing in their study populations. The first RCT concluded that perioperative pelvic floor muscle training reduced the risk of pelvic floor symptoms 12 weeks after surgery and improved quality of life. The second trial concluded that there was no significant benefit 12 months after surgery. However, when analyzing the results reported in this trial, urinary symptoms and quality of life also improved more in the treatment group. Perioperative PFMT may reduce the risk of pelvic floor symptoms and improve quality of life after pelvic organ prolapse (POP) surgery, although evidence is insufficient to implement it in current clinical practice. Abbreviations: PFMT: pelvic floor muscle training.

	Document Type	Objective/Focus	Evidence Base/Key Arguments	Critical Appraisal (JBI Domains)	Overall Appraisal
Lakeman M., et al. (2013) [54]	Clinical opinion/Expert commentary—Int Urogynecol J	To present the physiotherapist’s perspective on pre- and postoperative pelvic floor rehabilitation for women undergoing POP surgery, emphasizing clinical rationale and practical implementation.	The article provides expert interpretation of available literature, highlighting the need for early physiotherapy and PFMT before and after POP surgery to optimize outcomes and prevent recurrence. The reasoning is consistent and supported by reference to clinical studies and practice guidelines.	1. Source identified: Yes (authors with expertise in urogynecology and physiotherapy). 2. Standing in field: Yes. 3. Population focus: Yes (women undergoing POP surgery). 4. Evidence support: Partial (literature-based, not systematic). 5. Logical consistency: Yes. 6. Incongruities discussed: Yes (acknowledges limited data). 7. Based on relevant literature: Yes. 8. Overall appraisal: Include.	Credible and well-grounded clinical opinion; aligns with current evidence and physiotherapy standards. Moderate–high confidence.

## Data Availability

All relevant data are present within the manuscript.

## Supplementary Materials

The following supporting information can be downloaded at: https://www.mdpi.com/article/10.3390/jcm15031116/s1, Table S1: Preferred Reporting Items for Systematic reviews and Meta-Analyses extension for Scoping Reviews (PRISMA-ScR) Checklist.
